# Her2 amplification, Rel-A, and Bach1 can influence APOBEC3A expression in breast cancer cells

**DOI:** 10.1371/journal.pgen.1011293

**Published:** 2024-05-28

**Authors:** Madeline Dennis, Alyssa Hurley, Nicholas Bray, Cameron Cordero, Jose Ilagan, Tony M. Mertz, Steven A. Roberts

**Affiliations:** 1 School of Molecular Biosciences and Center for Reproductive Biology, Washington State University, Pullman, Washington, United States of America; 2 Department of Microbiology and Molecular Genetics, University of Vermont Cancer Center, University of Vermont, Burlington, Vermont, United States of America; Wayne State University, UNITED STATES

## Abstract

APOBEC-induced mutations occur in 50% of sequenced human tumors, with APOBEC3A (A3A) being a major contributor to mutagenesis in breast cancer cells. The mechanisms that cause A3A activation and mutagenesis in breast cancers are still unknown. Here, we describe factors that influence basal A3A mRNA transcript levels in breast cancer cells. We found that basal A3A mRNA correlates with A3A protein levels and predicts the amount of APOBEC signature mutations in a panel of breast cancer cell lines, indicating that increased basal transcription may be one mechanism leading to breast cancer mutagenesis. We also show that alteration of *ERBB2* expression can drive A3A mRNA levels, suggesting the enrichment of the APOBEC mutation signature in Her2-enriched breast cancer could in part result from elevated A3A transcription. Hierarchical clustering of transcripts in primary breast cancers determined that A3A mRNA was co-expressed with other genes functioning in viral restriction and interferon responses. However, reduction of STAT signaling via inhibitors or shRNA in breast cancer cell lines had only minor impact on A3A abundance. Analysis of single cell RNA-seq from primary tumors indicated that A3A mRNA was highest in infiltrating immune cells within the tumor, indicating that correlations of A3A with STAT signaling in primary tumors may be result from higher immune infiltrates and are not reflective of STAT signaling controlling A3A expression in breast cancer cells. Analysis of ATAC-seq data in multiple breast cancer cell lines identified two transcription factor sites in the *APOBEC3A* promoter region that could promote A3A transcription. We determined that Rel-A, and Bach1, which have binding sites in these peaks, elevated basal A3A expression. Our findings highlight a complex and variable set of transcriptional activators for A3A in breast cancer cells.

## Author summary

The protein APOBEC3A (A3A) is a major cause of mutations in breast cancers by changing cytidines in single-stranded DNA to uridine. The amount of A3A transcript is elevated specifically in cancer cells containing A3A-induced mutations, suggesting that up-regulation of A3A transcription may be an underlying cause of cancer mutations. However, little is known about the factors that influence A3A transcript level in breast cancer. Here, we identify three different proteins that impact A3A transcription: Her2, Rel-A and Bach1. Her2 activation is a common oncogenic event in some breast cancer subtypes and likely alters transcription of a variety of genes, including APOBEC3A, through enhanced signaling pathways or cell stress signaling. Contrastingly, Rel-A and Bach1 are transcription factors that we provide evidence that likely impact A3A transcription by binding the A3A promoter. Additionally, we found that correlations between A3A expression and canonical A3A transcriptional regulators, STAT1 and STAT2, in primary tumors stems from immune cells infiltrating into the tumor instead of direct transcriptional regulation in breast cancer cells. Our results demonstrate that A3A transcriptional regulation is highly complex with multiple factors and inputs potentially leading to an increase in mutations.

## Introduction

Mutation signatures consistent with the activity of apolipoprotein B mRNA-editing enzyme catalytic polypeptide-like (APOBEC) cytidine deaminases occur in approximately 50% of all sequenced human tumors [1,2]. These enzymes are part of the larger AID/APOBEC family that has a range of roles in the innate and adaptive immune response [3]. The APOBEC3 subset of proteins has a critical role in retroelement restriction [4] with several of the APOBEC3 proteins restricting HIV [5]. Additionally, some APOBEC3 proteins have off-target activity towards genomic DNA that can lead to oncogenic mutations [6,7], genome instability [8], and resistance to certain cancer therapies [9,10]. The mutations caused by APOBEC3 members are sequence specific: the cytidine in a TCW motif (where the W is an A or T base) is deaminated in ssDNA [4], leading to C to T, C to G, and a low level of C to A substitutions at TCW sequences, which produce the COSMIC single base pair substitution (SBS) signatures 2 and 13 in cancer genomes [11]. The APOBEC mutation signature is one of the more common among sequenced tumors [1], however SBS2 and SBS13 are enriched in certain cancers including breast cancer [11–13]. Of the 7 APOBEC3 proteins found in humans, APOBEC3A (A3A) likely contributes the majority of APOBEC-induced mutations in breast cancer [14,15], however, our understanding of the mechanisms that regulate APOBEC3A in breast cancer is inadequate.

Transcriptional upregulation could explain the significant contribution of APOBEC-induced mutations in tumors because elevated mRNA levels for APOBECs correlate with APOBEC-induced mutations and with genetic rearrangements in multiple cancer types [12,13]. However, transcriptional regulation of APOBEC3 proteins has been difficult to characterize due to all 7 proteins being expressed at various levels in many different tissues and cell types [16,17]. The most well characterized transcriptional response for APOBEC3A is in myeloid cells [17] with type I interferons (IFNs) acting as stimulators of expression for A3A [18]. Various external stimuli such as foreign nucleic acids [19,20], self-cytoplasmic DNA [21], poly (I:C) [22], and IL-27 [23] activate A3A expression via IFN signaling. Less is known about A3A transcriptional regulation in the context of cancer cells. APOBEC3B (A3B) mRNA levels are typically high in breast cancer [24], which has made it the focus of multiple studies [24–27]. A3B transcription is activated through different mechanisms including epidermal growth factor receptor (EGFR) signaling [28], estradiol signaling [29], and via various forms of NF-κB signaling [25,26]. Due to similarity in promoter regions, A3A expression could be elevated in breast cancer through similar mechanisms. The tumor-suppressor protein, p53, regulates both A3A and A3B in response to DNA damage [30]. However, genotoxic stress, possibly brought on by viral infection, and epidermal growth factor receptor (EGFR) signaling can transiently and uniquely induce A3A via the canonical NF-κB pathway [10,22]. Fluctuation in the activity of these signaling pathways, and as a result A3A expression, is thought to be one possible mechanism leading to episodic acquisition of A3A-induced mutations that has been reported in breast cancer cell lines [15,31]. The identity of the specific transcription factors and pathways that would cause episodic A3A transcription is still not fully understood.

Here, we evaluated specific proteins critical for basal A3A mRNA abundance in breast cancer cell lines using a combination of bioinformatics and cell-based experiments. We assessed A3A transcript levels in 20 breast cancer cell lines to correlate A3A expression and protein level with mutagenesis, showing that A3A transcript level is a primary determinant of protein abundance. We identify that Her2, Rel-A, Bach1, and to a lesser extent STAT1 containing complexes can influence the abundance of basal A3A transcript in breast cancer cells, with some effects being cell line specific.

## Results

### A3A transcript levels correlate with A3A protein level and pre-existing APOBEC-induced mutations

**Fig 1:**
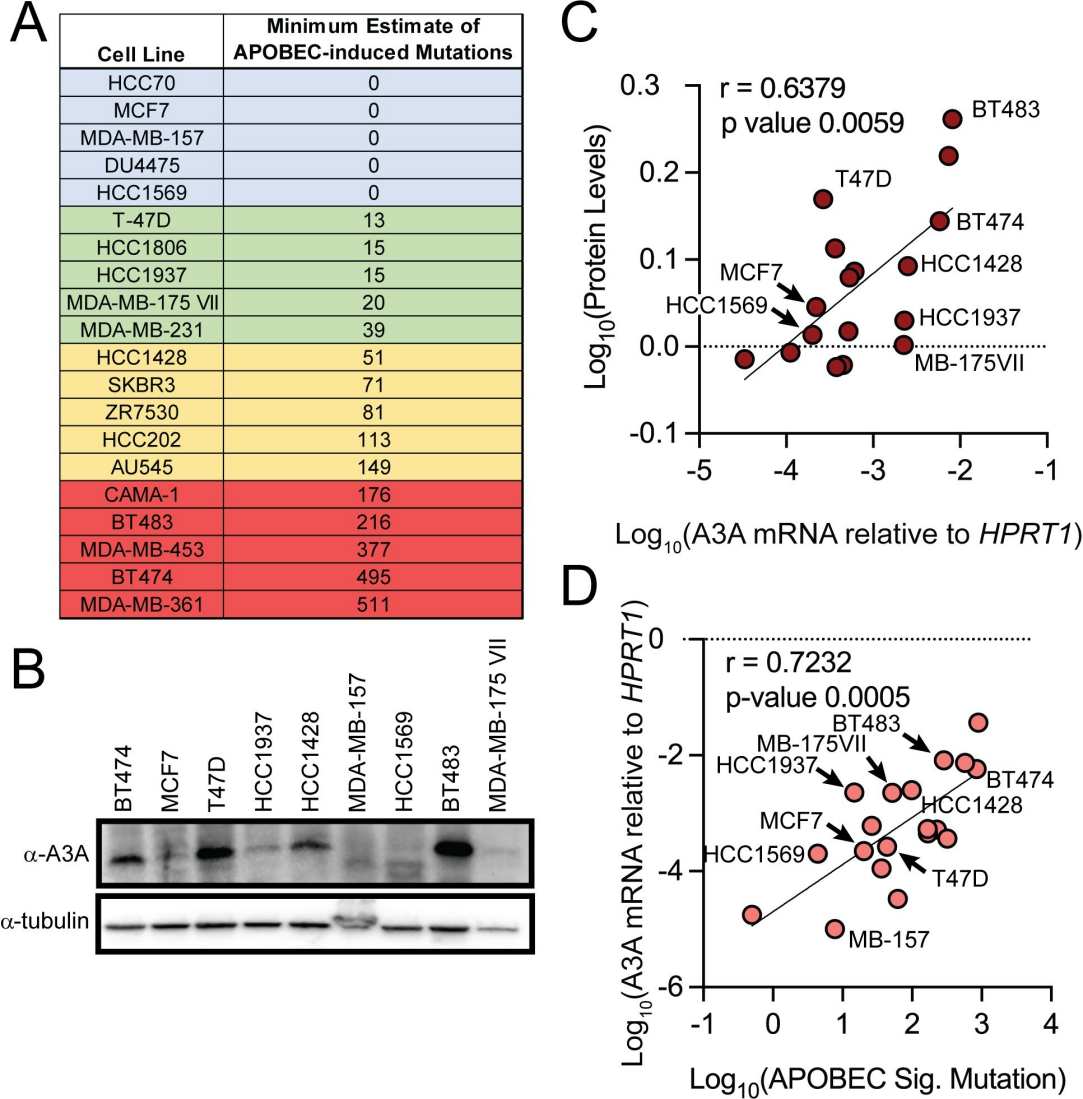
(A) Cells lines evaluated for A3A mRNA level, A3A protein level, APOBEC signature mutations, and cytidine deaminase activity. The estimated load of APOBEC-induced mutations in each cell line was determined as in [12]. Lines highlighted in blue, green, yellow, and red indicated those not mutated by APOBECs or containing low, moderate, and high amounts of APOBEC-induced mutations, respectively. (B) A representative western blot for A3A and ⍺-tubulin in 9 breast cancer cell lines. (C) Quantified A3A protein levels from breast cancer cell lines (as described in methods) including those in (B) correlated with A3A mRNA levels measured via qRT-PCR; Pearson coefficient of 0.6379. (D) A3A mRNA levels measured via qRT-PCR correlated with quantified SBS2/SBS13 mutations in cancer cell lines; Pearson coefficient of 0.7232. Cell lines shown in (B) are highlighted in correlation analyses in (C) and (D).

Transcriptional activation or repression are fundamental in determining the amount of cellular APOBEC protein and could influence how tumors accumulate APOBEC-induced mutations. APOBEC3A transcript levels correlate with APOBEC-induced mutations in sequenced primary breast cancer tumors [12,14]. We therefore evaluated APOBEC3A transcript levels (via qRT-PCR), protein abundance (by Western analysis), and number of APOBEC signature mutations (from publicly available mutation lists obtained from the Cancer Cell Line Encyclopedia [32]) in a panel of 20 breast cancer cell lines (Fig 1A) to establish the relationship between transcription and APOBEC mutations in a model system amenable to genetic assessment of the importance of specific factors to A3A expression. Correlation of steady-state A3A mRNA abundance and protein revealed a Pearson rho of 0.6379 (p-value = 0.0059), indicating that in breast cancer cell lines, A3A protein level is mostly predicated on transcription level (Fig 1B and 1C). Additionally, A3A transcript levels and A3A protein level positively correlates with the amount of cytidine deaminase activity across breast cancer cell lines [14] (S1 Fig), suggesting that A3A is responsible for most of the APOBEC activity in breast cancer cell lines and that the influences of post-translational regulation of A3A may be limited to specific contexts. A3A qRT-PCR data also correlated with the number of SBS2/SBS13 mutations that had been acquired in each cell line at the time of isolation from respective tumors, displaying a Pearson coefficient of 0.7232 (p-value = 0.0005) (Fig 1D). This data indicates that in cancer cell lines, like in primary breast adenocarcinoma tumors, transcript abundance of A3A is critical in determining its presence, which predicts the previous mutagenic activity of the protein.

### Her2 influences A3A transcript levels

**Fig 2:**
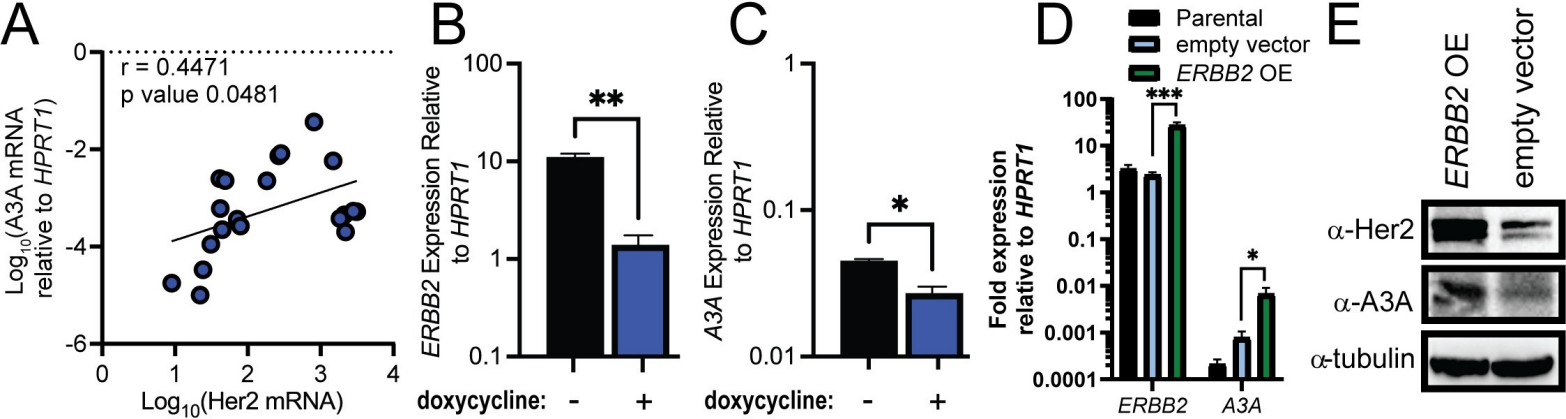
(A) Her2 transcript levels from RNA-seq data correlated with A3A mRNA levels measured via qRT-PCR; Pearson coefficient of 0.5447. (B) Her2 expression measured via qRT-PCR after shRNA knockdown with doxycycline inducible system. (C) A3A expression measured via qRT-PCR after doxycycline-induced *ERBB2* shRNA knockdown in BT474 cells. (D) *ERBB2* and A3A expression relative to *HPRT1* measured by qRT-PCR from parental, empty vector transfected, or *ERBB2* over-expressing (OE) T47D cells. All measurements show the mean value and standard deviation for 3 independent biological measurements. (E) Western blot analysis for Her2, A3A, and tubulin in empty vector transfected or *ERBB2* over-expressing (OE) T47D cells.

Her2-enriched breast cancers are enriched with APOBEC-induced mutations [12], indicating a possible relationship between Her2 amplification and A3A. We correlated *ERBB2* RNA-seq data from The Cancer Cell Line Encyclopedia [32] with the A3A transcript levels measured by qRT-PCR and found a moderate correlation with a Person Rho coefficient of 0.4471 (p-value = 0.048) (Fig 2A). Based on this correlation, we reduced Her2 in BT474 cells to elucidate its impact on basal A3A expression. We transduced these cells with a doxycycline inducible *ERBB2*-targeting shRNA construct. Addition of doxycycline to express the *ERBB2* shRNA resulted in a maximal 10-fold decrease in Her2 expression due to BT474 dependence on Her2 causing cell death. Nevertheless, the 10-fold decrease in Her2 correlated with a 4-fold reduction in A3A mRNA abundance (Fig 2B and 2C), indicating that upstream Her2 signaling can influence A3A transcription. In converse to BT474 cells, T47D breast cancer cells lack *ERBB2* amplification and have low levels of A3A mRNA. We therefore transiently transfected these cells with an *ERBB2* over-expression vector to increase Her2 levels. This transfection increased *ERBB2* mRNA greater than 10-fold compared to an empty vector control (Fig 2D). In concert, A3A mRNA levels increased 7-fold with higher Her2 expression. Both impacts of Her2 over-expression on *ERBB2* and A3A mRNA translated to protein abundance (Fig 2E), suggesting that Her2 amplification and enhanced signaling can increase A3A expression in some breast cancer cells.

### A3A co-expression with immune signaling genes in primary breast tumors is likely due to infiltrating immune cells

**Fig 3:**
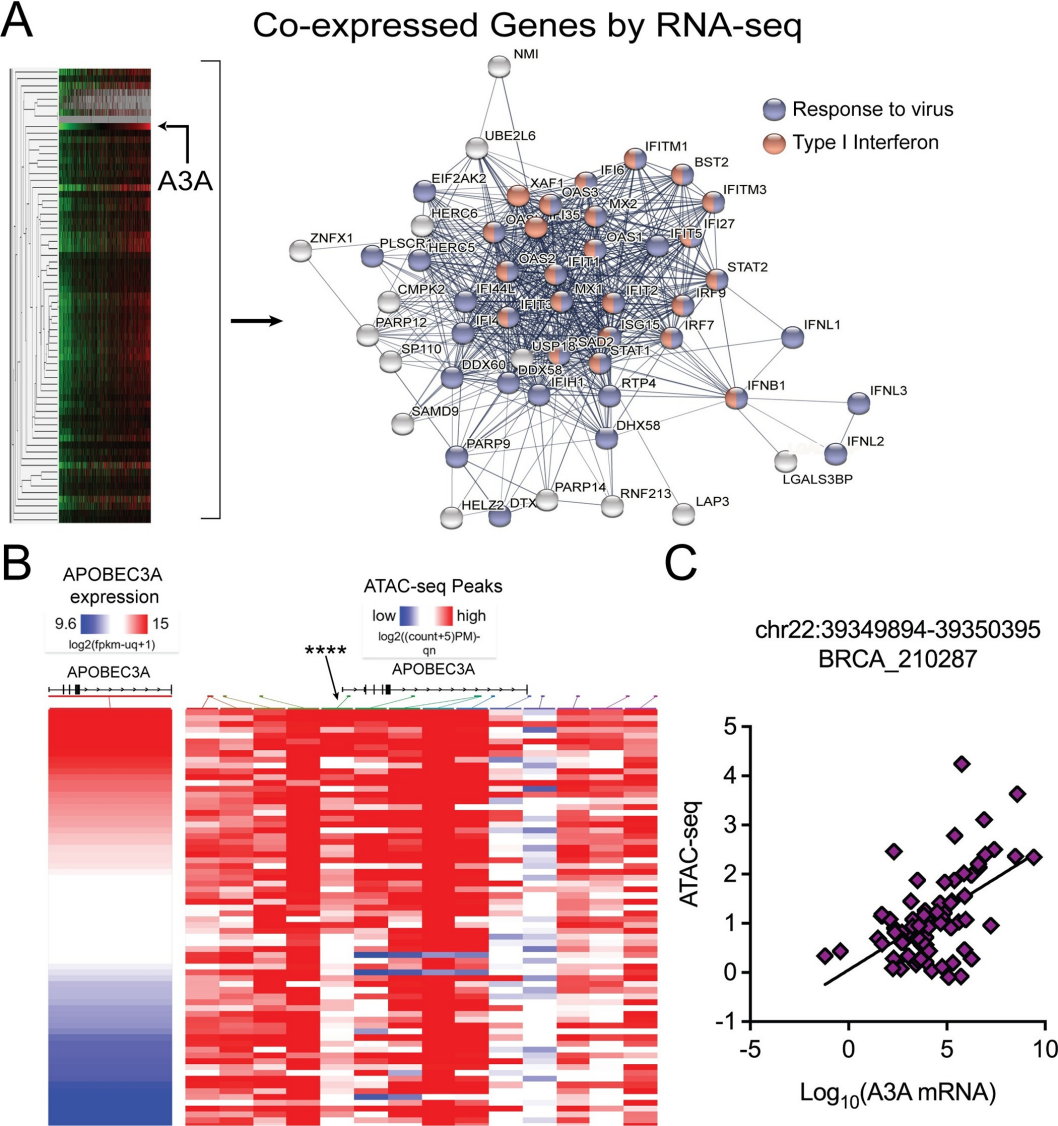
(A) Network of genes co-expressed with *APOBEC3A*. Hierarchical clustering of breast cancer tumor RNA-seq data from The Cancer Genome Atlas (TCGA) produced a node of 66 co-expressed genes. Gene ontology analysis of co-expressed genes highlighted “response to virus” (purple spheres) and “type I interferon signaling” (red spheres) as enriched processes. (B) Heatmap of ATAC-seq peak heights from 75 breast cancer tumors organized in terms of increasing A3A expression. **** indicates a peak whose presence correlates with the A3A pattern of expression among tumors. The location of the ATAC-seq peaks near the APOBEC3 loci on chr. 22 relative to the *APOBEC3A* gene is indicated by colored arrows and blocks stemming from each column. Relative expression levels and ATAC-seq peak heights are indicated at a heat map with red indicating high expression and open chromatin and blue representing low expression and more closed chromatin. (C) A3A mRNA data was correlated with the ATAC-seq peak reads for the location of Chr22: 39349894–39350395.

We next obtained RNA sequencing data from The Cancer Genome Atlas (TCGA) [33] and identified 66 genes that are expressed similarly to A3A in primary breast cancer tumors by hierarchical clustering (Fig 3A and S1 Table). We conducted gene ontology (GO) analysis on the set of genes to determine enriched cellular processes among the genes that are expressed similarly to A3A. Not unexpectedly, most genes were related to immune responses, specifically response to virus (purple spheres) and type 1 interferon signaling (red spheres) (Fig 3A). Additionally, we utilized transposase-accessible chromatin (ATAC-seq) data from 75 BRCA tumors in the TCGA database [33] to find open chromatin areas in the *APOBEC3A* promoter region that associate with higher A3A mRNA levels and are therefore likely to contain binding sites for transcription factors regulating A3A expression. Correlating ATAC-seq peak heights with A3A expression levels identified several peaks significantly associated with A3A mRNA level (Fig 3B). The strongest associating peak was located at coordinates of Chr22: 39349894–39350395. The site (indicated with ****) correlates strongly with the expression of A3A in breast cancer tumors (Fig 3C). Additionally, this site encompasses previously identified signal transducer and activator of transcription 2 (STAT2) and Rel-A binding sites (transcription factor binding sites obtained from The Gene Transcription Regulation Database [34]) that is responsive to viral infection and DNA damage, respectively [22]. We were particularly interested in the type 1 interferon signaling pathway because A3A is typically regulated via type 1 interferon signaling in immune cells (reviewed in [18]). The clustering results also identified STAT1 and STAT2, two critical transcription factors mediating type 1 interferon signaling [35], were typically co-expressed with A3A. Direct comparison of the STAT1 and STAT2 transcript abundance with that of A3A in primary breast cancers revealed strong positive correlations with Pearson’s rho values of 0.42 and 0.36, respectively (S2 Fig), suggesting these factors could modulate A3A transcription during breast cancer development.

**Fig 4:**
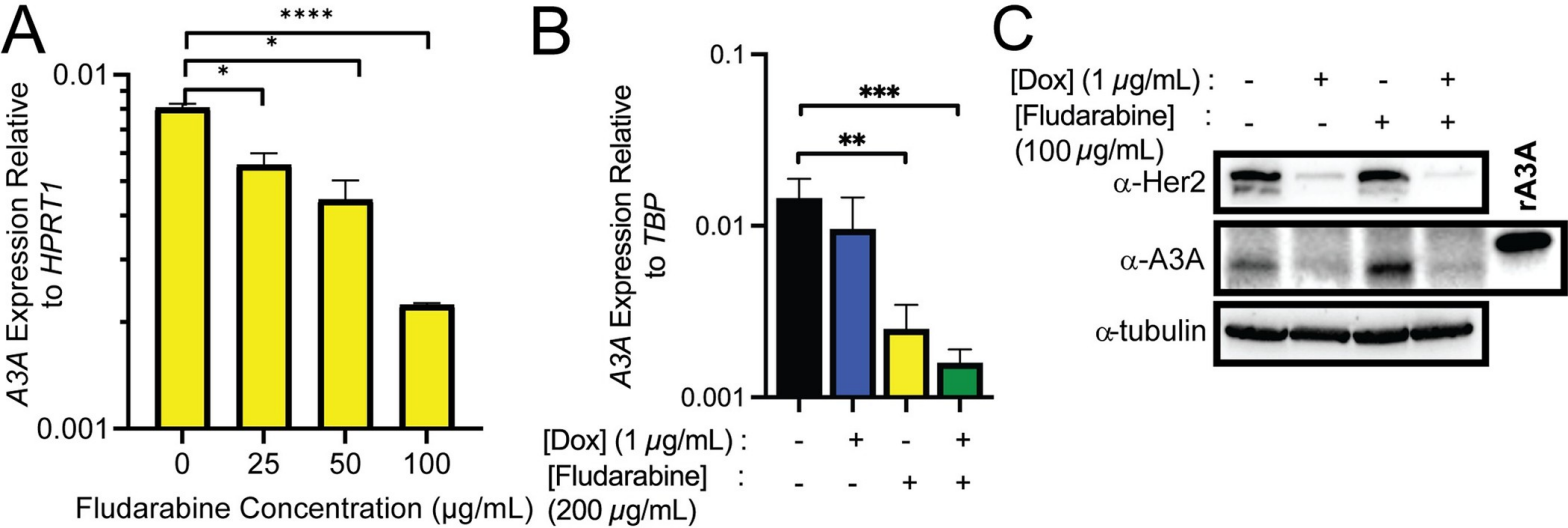
(A) A3A mRNA levels measured via qRT-PCR after 72-hour treatment of BT474 cells with various concentrations of fludarabine. (B) Double treatment of doxycycline to induce Her2 shRNA and fludarabine to block STAT signaling in BT474 cells. A3A mRNA levels measured via qRT-PCR. All measurements show the mean value and standard deviation for 3 independent biological measurements. (C) Western blot analysis of Her2, A3A, and tubulin levels in doxycycline-induced Her2 shRNA knockdown and fludarabine treated BT474 cells.

To determine if type 1 interferon signaling impacted A3A expression levels in breast cancer cells, we initially treated BT474 cells with fludarabine for 72 hours and then harvested cells for qRT-PCR analysis. Fludarabine is a nucleotide analog that prevents the activation of STAT1 as well as inhibits DNA synthesis [36,37]. We found that A3A expression was decreased approximately 4-fold with the highest concentration of fludarabine (Fig 4A). Since we observed a moderate decrease in A3A mRNA with both *ERBB2* knockdown and fludarabine treatment, we attempted to further reduce A3A expression with a combinatorial treatment of doxycycline-induced *ERBB2* knockdown and 200 μg/mL fludarabine (the highest concentration before the drug induces cell death). As shown in Fig 4A, we found a significant reduction in A3A mRNA with fludarabine alone. However, the combined treatment of *ERBB2* knockdown and fludarabine produced an additive effect, resulting in a ~10-fold reduction in A3A transcript levels (Fig 4B). *ERBB2* knockdown itself or in combination with fludarabine also reduced A3A protein level, however, the impact on A3A protein appeared to be mostly provided by *ERBB2* reduction (Fig 4C). This combinatorial reduction implies that multiple pathways influence basal A3A transcription, however, the mechanism by which this occurs is still unknown.

We next wanted to assess the generality of fludarabine inhibition of A3A mRNA across multiple breast cancer cell lines. We therefore treated AU565, T47D, and HC1395 cells with 25 to 200 μg/mL fludarabine for 72 hrs prior to assessing A3A mRNA. Fludarabine failed to significantly alter A3A mRNA level in any of these lines, indicating the drug’s impact on A3A is limited to BT474 cells (S3A Fig). We therefore attempted to confirm that fludarabine treatment reduced A3A expression through its inhibition of STAT1/2 complexes since fludarabine could have off-target effects. Since *APOBEC3A* is canonically regulated by STAT2 signaling in immune cells [38], we focused on reducing STAT2 expression in BT474 cells with an shRNA construct. This STAT2-targeting shRNA reduced STAT2 mRNA abundance 82%. A3A expression, however, remained unchanged (S4 Fig), indicating that fludarabine’s impact on A3A mRNA in BT474 is possibly due to an unknown off-target mechanism or a STAT1 homodimer complex. We also evaluated STAT1 and STAT2 impacts on A3A mRNA in MDA-MB-453 breast cancer cells. In this cell line, respective shRNAs reduced STAT1 expression 94% and STAT2 expression 91% (S3B and S3C Fig). In contrast to BT474 cells, these reductions in STAT1/2 were accompanied by respective 5- and 11-fold reductions in A3A mRNA, indicating that STAT1/2 signaling may impact A3A expression, but only in select cell lines.

STAT1/2-mediated transcriptional changes usually involve activation of STAT1 and/or STAT2 by their phosphorylation by a Janus kinase (JAK). To gain a more general assessment of STAT signaling on driving basal A3A expression in breast cancer cell lines, we therefore profiled STAT1, phospho-STAT1, STAT2, phosphor-STAT2, and A3A abundance by western analysis across 7 breast cancer cell lines (S5A Fig). Basal levels of phospho-STAT1 and phospho-STAT2 across these lines was extremely low, and in many cases undetectable, despite consistent STAT expression and variable A3A expression across the evaluated cell lines, suggesting that STAT phosphorylation has a limited role in determining A3A protein level. We next directly tested the role of STAT phosphorylation in promoting basal A3A mRNA level by treating BT474 and MDA-MB-453 cells with concentrations of the JAK-inhibitors, ruxolitinib and pacritinib, at concentrations previously determined to block STAT-mediated induction of A3A [22] (S5B Fig). Addition of these inhibitors to cell media resulted in little to no change in A3A mRNA, further supporting a limited role of STAT signaling in basal A3A expression in breast cancer cells.

**Fig 5:**
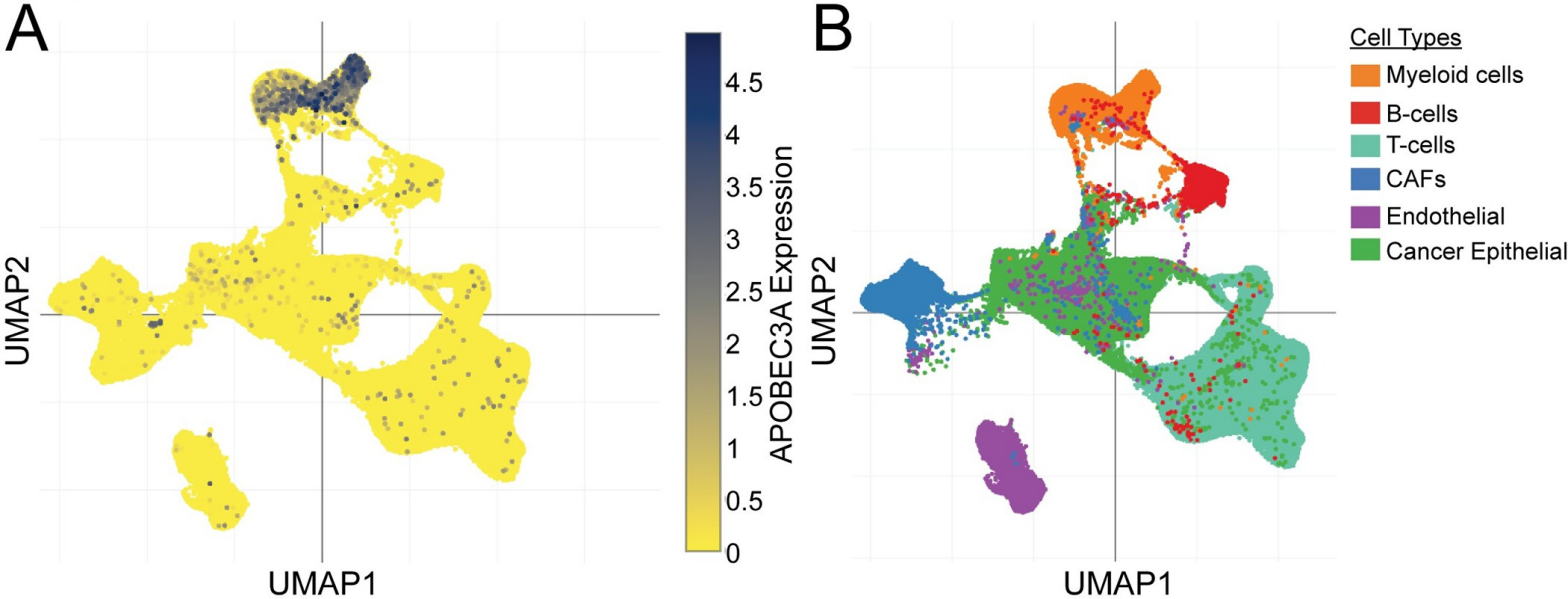
Single cell RNA sequencing data from primary breast cancer tumors. (A) A3A transcript levels in individual cells of primary breast cancers. Different cell types within the tumor are presented in (B) and indicated by color with myeloid cells (orange), B-cells (red), T-cells (light blue), cancer-associated fibroblasts (CAFs; dark blue), endothelial cells (purple) and cancer epithelial cells (green) depicted.

The disconnect existing between the strong correlation of A3A mRNA levels with STAT1/2 signaling in primary tumors and the generalized lack of an impact on STAT1/2 on A3A transcription in breast cancer cell lines prompted us to assess whether the heterogenous cell types within a tumor may be influence correlations observed using bulk RNA-seq tumor data. We therefore interrogated publicly available single cell (sc) RNA-seq data from primary breast cancer samples [39] to determine the A3A mRNA level in different cell types within tumors. The cells resident within 26 breast cancer samples were categorized by expression of cell type specific markers and visualized spatially utilizing a UMAP plot (Fig 5). Overlay of A3A expression level within these cells revealed A3A was most highly and uniformly expressed within infiltrating immune cells in the individual tumors. A3A mRNA level within breast cancer cells themselves was detectable, but at significantly lower level, indicating that the primary factor dictating A3A expression level in bulk RNA-seq analyses is likely the amount of immune cell infiltration instead of the expression level within the tumor cells themselves. This confounding factor likely produces the strong correlations between A3A expression and immune signaling within bulk RNA-seq analyses, while STAT1/2 signaling likely plays a more limited role in dictating A3A expression levels in breast cancer cells. While A3A levels are lower in breast cancer cells, they are still likely sufficient to induce mutagenesis as CRISPR-mediated disruption of the APOBEC3A gene results in a loss of APOBEC signature mutations in BT474 and MDA-MB-453 breast cancer cell lines [15].

### ATAC-seq shows critical regulation elements in cell lines

**Fig 6:**
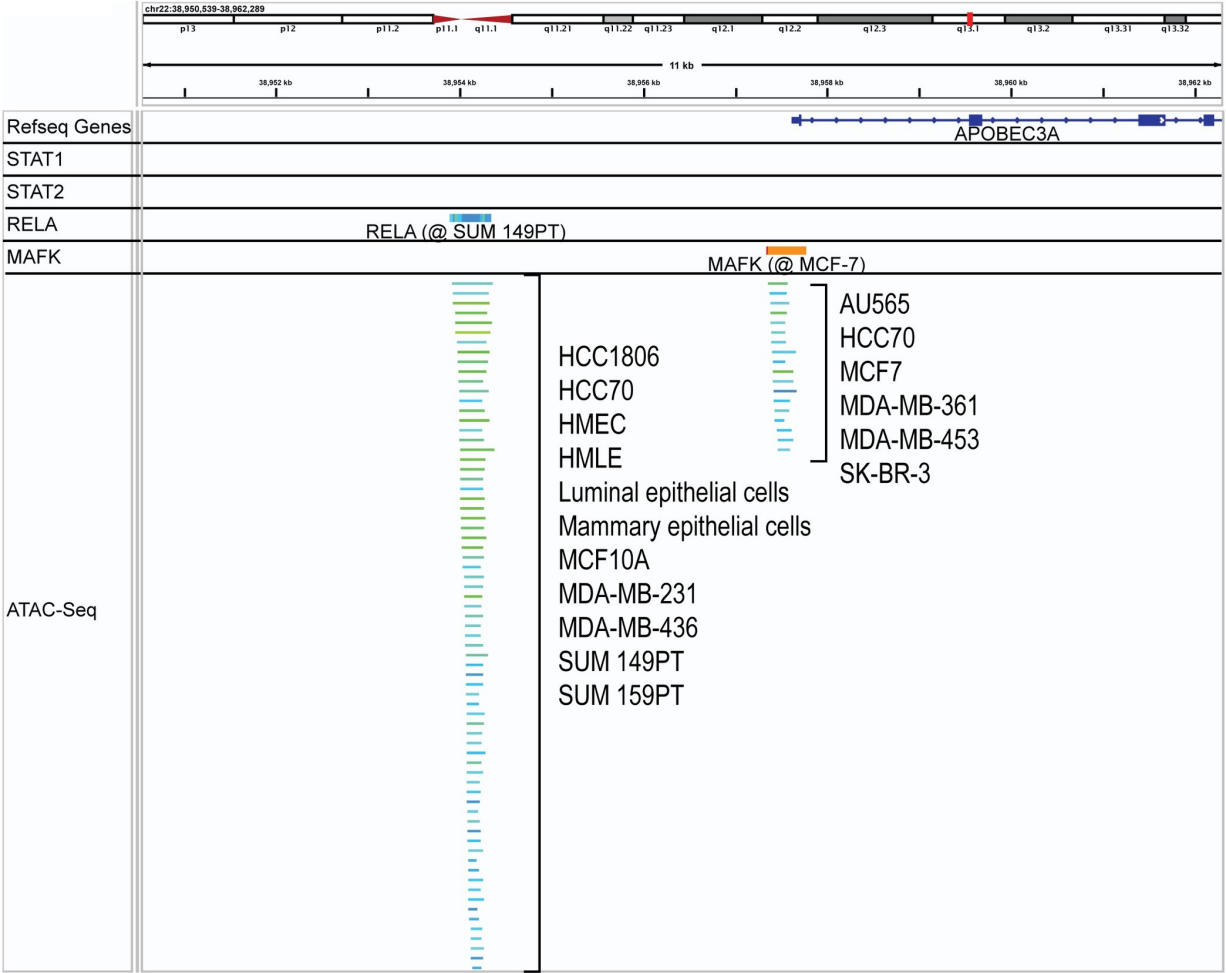
ATAC-seq data from various breast cancer cell lines aligned near the *APOBEC3A* gene with two peak sites. Cell lines in which peaks were observed are listed. ATAC-seq peaks overlap with a ChIP-seq defined Rel-A binding site (3 kb upstream of the TSS) and a MAFK binding site (TSS proximal ATAC-seq peak). STAT1 nor STAT2 binding was observed.

We next attempted to identify other potential regulators of A3A expression within breast cancer cells specifically. We analyzed ATAC-seq peak calls from multiple breast cells obtained from the ChIP-Atlas [40, 41]. Among these lines, two peaks occurred within the A3A promoter (Fig 6). One peak, approximately 3 kb upstream of the transcription start site (TSS), corresponds to the same location identified in the tumor data as correlating with A3A expression and contains a Rel-A binding site (Fig 6). The other peak, located proximal to the A3A TSS, occurred in AU565, SK-BR-3, MDA-MB-361 and MDA-MB-453, which contain SBS2/13 mutations, in addition to MCF7 and HCC70 cells. This peak also contained a binding site for Rel-A, but additionally contained multiple binding sites for MAF proteins (S2 Table). Small MAF proteins consist primarily of a DNA binding domain that typically dimerizes another transactivating protein to regulate downstream expression of target genes. In many cases, the transactivating partner is Bach1 or Bach2, with Bach1 being heavily implicated in breast cancer metastasis [42]. ChIP-seq analysis of Rel-A and MAFK in breast cancer cell lines found peaks for both proteins, with Rel-A binding specifically at the ATAC-seq peak 3 kb upstream of the *APOBEC3A* TSS, while MAFK bound at the ATAC-seq peak proximal to the TSS, supporting that these open chromatin sites were likely functional in regulating A3A expression by direct binding of these transcription factors at the respective transcription factor binding sites.

### Rel-A and Bach1 elevate basal A3A expression in BT474

**Fig 7:**
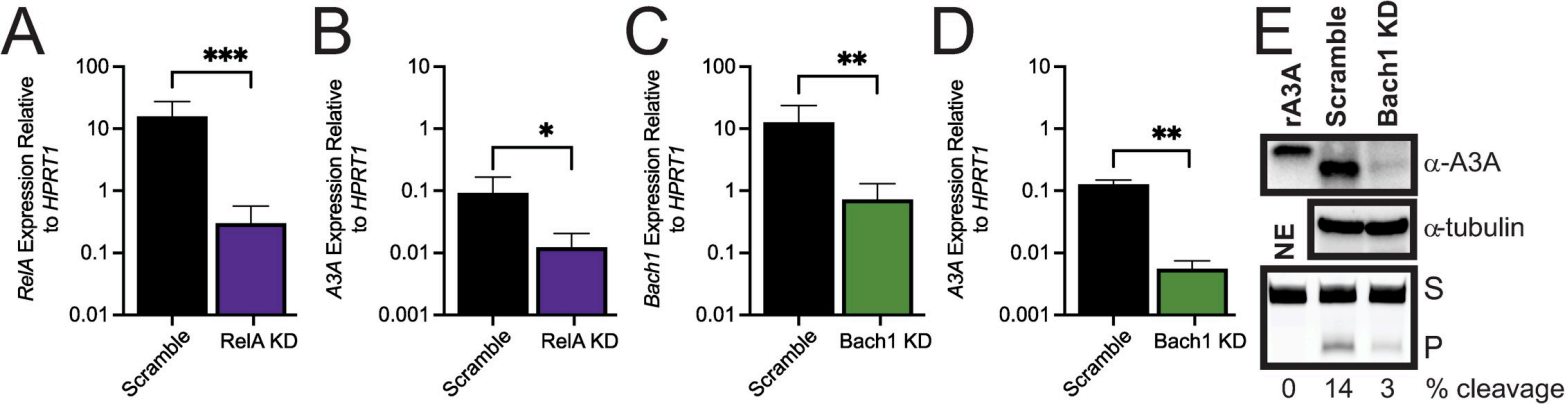
(A) Rel-A and (B) A3A expression measured via qRT-PCR after Rel-A shRNA knockdown in BT474 cells; scramble = non-targeting shRNA, KD = Rel-A-targeting shRNA. (C) Bach1 and (D) A3A expression measured via qRT-PCR after Bach1 shRNA knockdown in BT474 cells; scramble = non-targeting shRNA, KD = Bach1 targeting shRNA. All measurements show the mean value and standard deviation for 3 independent biological measurements. (E) Western analysis of A3A and tubulin protein levels in scramble control and Bach1 knockdown BT474 cells. Cellular cytidine deaminase activity (lowest panel) was also determined by in vitro cytidine deaminase activity assay using a hairpin oligonucleotide substrate. NE = no extract, S = Substrate, P = product.

We next assessed the contribution of Rel-A to A3A basal expression because it has multiple potential binding sites within the A3A promoter region, induction of A3A can be mediated through Rel-A [22], and NF-κB signaling contributes to the expression of other APOBECs [25,26,43]. We therefore utilized shRNA to reduce Rel-A mRNA 98% in BT474 cells (Fig 7A). This reduction in Rel-A correlated with a 7.5-fold reduction in A3A expression (Fig 7B). Similarly, reduction of Rel-A in MDA-MB-453 cells resulted in a 26-fold reduction in A3A mRNA (S3D Fig), indicating that NF-κB signaling likely plays a more general role in regulating A3A expression in breast cancer cells than STAT1/2. Considering that the TSS-proximal *APOBEC3A* promoter ATAC-seq peak contained multiple MAF binding sites and that all the small MAFs can dimerize with Bach1/2 to mediate gene transactivation, we decided to determine if Bach1 can regulate A3A expression. This possibility was further supported by the identification of Bach1 as a potential regulator of *APOBEC3A* by the Predicting Associated Transcription factors from Annotated Affinities (PASTAA) software [44]. We input genes co-expressed with A3A from TCGA profiled breast cancers (used in for GO analysis in Fig 3) into PASTAA to identify potential transcription factors that may globally regulate this gene set. The v-MAF transcription factor binding site (which includes sites for small MAF proteins) was indicated as a likely regulator of *APOBEC3A* expression. Moreover, PASTAA specifically indicated A3A as a potential Bach1 target (S3 Table). We therefore knocked down Bach1 in BT474 cells with a shRNA and reduced its expression 94%. Assessing A3A mRNA level in these cells by qRT-PCR revealed a 17-fold reduction of A3A expression, which is the most significant reduction in A3A mRNA level that we found in BT474 cells (Fig 7C and 7D). In concert with A3A expression, reduction of Bach1 in BT474 cells also reduced A3A protein levels to nearly undetectable levels (Fig 7E), suggesting that binding of a MAFK-Bach1 complex at the A3A promoter is promoting A3A mRNA expression and controlling A3A protein abundance in human breast cancer cells. Steady-state Bach1 and A3A mRNA levels in breast cancer cell lines, however, do not significantly correlate (S6 Fig), indicating that Bach1 influence on A3A transcription occurs through Bach1 protein level and is independent of Bach1 mRNA abundance.

## Discussion

Here, we identified multiple proteins that impact A3A transcription to varying extents in breast cancer cell line models. Knockdown or overexpression of Her2 reduced and increased A3A transcript levels in BT474 (Her2-enriched) and TD47 (low *ERBB2* expression) breast cancer lines. The ATAC-seq data analyzed from tumors and cell lines identified two main peaks within the *APOBEC3A* promoter region containing multiple STAT1/2, Rel-A, and MAF transcription factor binding sites. Reduced expression of Rel-A and Bach1 (a common small MAF binding partner) significantly reduced basal A3A mRNA expression levels, with Bach1 producing the largest decrease in the cell line BT474. STAT signaling however, only impacted A3A expression in one cell line, despite strong correlations between immune signaling and A3A mRNA in primary tumors. These correlations likely stem from high A3A expression in infiltrating tumor immune cells where STAT1/2 would be expected to be the primary A3A regulators. In contrast, our cell line data indicate that Rel-A and small MAF transcription factors whose transactivation activity is mediated by Bach1 are potentially the major transcription factors controlling A3A expression in breast cancer cells.

### Correlation of A3A expression and APOBEC-induced mutations in breast cancer cell lines

Correlation between A3A mRNA abundance and the number of APOBEC signature mutations in primary human tumors [14] suggests that increased transcription of *APOBEC3A* is a major factor contributing to these mutations. Moreover, changes in *APOBEC3A* expression during tumor development is thought to be a likely reason for the reportedly episodic nature of APOBEC signature mutation accumulation [31]. A highly variable level of A3A would seem to predict that the signaling pathways that upregulate *APOBEC3A* expression to its highest level would most readily predict the extent of A3A-induced mutations. We show here however that in breast cancer cell lines lacking external signals that upregulate *APOBEC3A* expression that the basal level of A3A mRNA correlates very strongly with the number of APOBEC signature mutations that were accumulated during the prior histories of the cell lines’ derivation. Since the mutations in these lines were acquired long before we assessed basal transcription levels, such a relationship between mutation number and mRNA or protein abundance indicates that a sustained upregulation of A3A over the lifetime of the tumor cell may be important for mutagenesis. Alternatively, variations in A3A mRNA could occur regularly resulting in averaged mutation accumulation over time correlating with the average A3A expression within a population of cells. In either case, the processes that influence A3A mRNA abundance during breast cancer development are essential for establishing APOBEC signature mutations.

### Multi-factor regulation of APOBEC3A

While we have identified several signaling and transcription factors (i.e. Her2, Rel-A, and Bach1) whose expression influences A3A mRNA abundance, the mechanisms for how each transcription factor controls A3A is still unclear. In certain cases, the transcription factors may directly interact with *cis* elements of the A3A promoter and activate transcriptional activation of the A3A gene. This is likely the case for Rel-A because it has been shown to interact with the A3A promoter to induce A3A expression during viral infection and DNA damage response [22]. Conversely, the proteins could be acting upstream of other important regulators that are in many cases yet to be determined. The transcriptional regulator downstream of Her2 signaling for A3A is currently unknown. However, recent evidence has been obtained indicating that *ERBB2* amplification can alter oesophageal cancer transcription through the activity of AP1 transcription factors [45]. AP1 binding sites are present in the APOBEC3A promoter indicating this mechanism may be relevant to A3A expression. It is also still unknown if Bach1 is directly affecting A3A mRNA levels by binding to the promoter region; it is possible that Bach1 is simply regulating other genes that activate A3A transcription.

### Cell specific regulation of APOBEC3A in breast cancers

In the cases of fludarabine treatment and STAT1/2 knockdowns, we identified cell specific transcriptional regulation of APOBEC3A, where application of the STAT1 inhibitor resulted in lower APOBEC3A mRNA abundance in BT474 cells but had no impact in other cell lines tested and STAT2 inhibition primarily decreased A3A expression in MDA-MB-453 cells. These results indicate that APOBEC3A transcriptional regulators may function only in cell specific contexts. What signaling response defines the spectrum of factors regulating APOBEC3A expression in breast cancers is unknown. Also unknown is how broadly Bach1 impacts A3A expression. Bach1 may have a greater likelihood of having a direct and conserved response across cell lines due to its altered functionality as a transcriptional activator during breast cancer metastasis. The variability in APOBEC3A transcriptional regulation between cell lines likely occurs at the promoter transactivation level. This is evident in the ATAC-seq data from Fig 6; even though there are two areas that have the same peak in multiple cell lines, not every cell line displays these peaks. This implies that if the critical transcription factor binds there, it may have a more pronounced effect in one cell line over another.

### Impacts of tumor heterogeneity on understanding A3A transcriptional regulation

Understanding the mechanisms that control A3A expression is critical as A3A transcription predicts the extent of APOBEC-induced mutations in a tumor, which have been shown to effect patient outcomes [8,27,46–48], therapy resistance, and tumor relapse [10,49]. Nevertheless, experimental validation of these factors’ impact on A3A has been difficult because relationships between genes identified as potential A3A regulators in primary tumors are not necessarily reproducible in cell lines. One possible reason for this discrepancy is that most RNA sequencing of tumors includes bulk cell populations and therefore lack differentiation between cancerous cells and stromal cells within the tumor. High expression of specific genes within the supporting cell matrix of the tumor can influence correlations and not be necessarily relevant to co-expression in cancer cells. This is evident in the co-expression of STATs and A3A from RNA-seq of primary breast cancers. We are unable to see a consistent effect from inhibiting STATs in cell lines despite strong correlations between STAT1/2 and A3A mRNA levels in primary cancer samples. Rather than affecting basal A3A expression, STAT1/2 could promote A3A expression transiently in response to specific stimuli. Additionally, analysis of single cell RNA sequencing data of primary breast cancers [39] indicates that the strongest A3A expression occurs in infiltrating myeloid cells (the typical cell type that A3A is expressed in [18]) with lower A3A expression in the cancerous cells that are accumulating the APOBEC-induced mutations (Fig 5). This suggests that the correlations we draw from bulk RNA sequencing data from primary tumors may not necessarily apply to the cancer cells and highlights the importance of future analyses to take cellular heterogeneity into account through the use of single-cell RNA sequencing technologies.

## Materials and methods

### Cell culture conditions

AU565, BT474, CAMA1, HCC70, HCC202, MCF7, MDA-MB361, MDA-MB-453, SKBR3, T47D, HCC1806, HCC1428, ZR7530, HCC1937, MDA-MB-231, MDA-MB-175 VIII, BT483, HCC1395, and HCC1596 cells were obtained from ATCC and cultured with recommended conditions (https://www.atcc.org/). Each cell line was identified and tracked using cell morphology characteristics provided by ATCC.

### Construction of Lentiviral shRNA vectors and *ERBB2* overexpression vector

Oligonucleotides (ordered from Integrated DNA Technologies; listed in S4 Table) were phosphorylated with T4 Polynucleotide Kinase (NEB M0201S) and annealed. The lentiviral vectors pLKO.1 or pLKO.1_TET (both marked with Hygromycin B) were used to clone the annealed oligos into with T4 DNA Ligase (NEB M0202M). *ERBB2* was PCR amplified from pBABEpuro-ERBB2 (Addgene #40978) using T4 PNK treated forward oligo (oACH_001) and a reverse oligo (oTM-795). The resulting PCR product was digested with NotI and inserted into pcDNA3β (gift from Ralph Scully) using EcoRV and NotI cut sites. The plasmids were transformed into Stbl3 *E*. *coli* cells (ThermoFischer, C737303) and selected for on LB + Carbenicillin plates. 5mL cultures of single colonies from plates were grown and plasmids extracted with Omega Biotek E.Z.N.A. Plasmid DNA Mini Kit I (D6942-00S). Plasmid sequences were verified via Sanger sequencing or whole plasmid sequencing performed by Plasmidsaurus using Oxford Nanopore Technology with custom analysis and annotation.

### Lentiviral transductions

Lentiviral shRNA plasmids with packaging plasmids psPAX2 (Addgene, #12260) and pMD2.G (Addgene, #12259) were co-transfected into HEK293T cells with viafect reagent (Promega E4981) to generate lentivirus. 48 hours following transfection, the lentiviral supernatant and Lentiblast transduction reagent (OZ biosciences) (diluted 1:250 in lentiviral supernatant) were added to either BT474 or MDA-MB-453 cells. After 24 hours, the lentiviral supernatant was removed. Stable cell populations were selected by the addition of HygromycinB at 500 μg/ml (BT474) and 200 μg/mL (MDA-MB-453) at 72 hours post-transduction.

### Breast cancer cell line transfections

T47D cells were seeded 500,000 cells per well in 6-well plates 24 hours before transfection with the *ERBB2* overexpression plasmid using Lipofectamine 3000 (Invitrogen). 2.5ug DNA, 5uL p3000 reagent and 7.5uL Lipofectamine 3000 reagent were used per well (according to Invitrogen’s cell-line specific recommendations). Cells were harvested for RNA isolation 72 hours-post transfection.

### Cell treatments

Cell lines were treated with various concentrations of fludarabine (APExBio catalog # A5424 or MedChemExpress catalog #HY-B0069) for 72 hours in normal culturing conditions before harvesting cell pellets. Doxycycline was used at 1 μg/mL for 72 hours to induce shRNAs. Cell pellets were subsequently processed for RNA isolation. For JAK inhibitor experiments, BT474 and MDA-MB-453 cells were treated with 2 μM of Pacritinib (MedChemExpress Catalog #HY-16379) or 2 μM of Ruxolitinib (MedChemExpress Catalog #HY-50856) for 16 hours and then harvested for RNA isolation.

### qRT-PCR

Cells were harvested during the log-phase of growth around 70% confluency. The cells were passed through a homogenizer column and total RNA was extracted with Omega Biotek Total RNA Kit II. cDNA was generated by combining 2 μg of DNAseI (ThermoFisher)-treated RNA with the cDNA reaction mixture (2.5 μM oligo dT23VN and 3.5 μM random hexamers, 0.5 mM dNTPs, 1x Mashup RT Reaction Buffer (25mM Tris-HCl pH 8.3, 25 mM MOPS pH 7.9, 60 mM KCl, 4 mM MgCl_2_, 5% glycerol, 0.006% IGEPAL CA-630), 10 mM DTT, 0.5 μL Mashup Reverse Transcriptase and 16 U RNase Inhibitor (Ribolock Rnase Inhibitor, ThermoFisher)) in a total volume of 20 μL. Reactions were incubated sequentially at 25°C for 5 minutes, 42°C for 60 minutes, and finally 65°C for 20 minutes prior to storage at -20°C. 2 μL cDNA was then combined with 10 μL of Forget-Me-Not Eva-Green 2X Master Mix, 0.5 μL of 10 μM primer (S4 Table), and 7 μL of sterile water. qPCR amplification reactions were run on a Bio-Rad CFX96 with the following protocol: incubate at 95°C for 5 min and followed by 40 cycles of 95°C for 10 s and 62.5°C for 1 min. Melt curves were performed to confirm that singular products were generated for all reactions.

### Western blots

20 or 50 μg of protein from cell extracts made in either RIPA or M-PER buffers for STAT1, STAT2, Phospho-STAT1, Phospho-STAT2, Her2, A3A, and tubulin blots was run on pre-made Mini-Protean TGX gels (Bio-Rad) at 165 V for 30–35 minutes or 140 V for 60 minutes in 1X TGS buffer (25mM Tris, 192mM Glycine, 0.1% SDS). Gels were transferred onto either a 0.2 μm PDVF membrane (Thermo scientific) or 0.45 μm nitrocellulose membrane (BioRad) via a Trans-Blot turbo transfer system (BioRad) or submerged tank transfer system (BioRad) in transfer buffer (25mM Tris, 192mM Glycine, 20% methanol, 0.01% SDS). STAT1, STAT2, Phospho-STAT1 and Phospho-STAT2 westerns were transferred using the turbo transfer mixed-MW setting. A3A westerns were transferred using the low MW setting or with the submerged tank for 1 hour at room temperature. ⍺-tubulin westerns were transferred using the standard MW setting or with the submerged tank transfer. Membranes were blocked in 2% ECL prime blocking agent (Cytiva) or 5% milk in filtered TBS-T (20mM Tris, 150mM NaCl, 0.1% Tween-20). Blots were incubated with the following antibodies in 1% ECL in 1X TBS-T: anti-STAT1 (Sigma Aldrich 06–501; 1:1000), anti-STAT2 (Cell signaling 72604, 1:1000), anti-phospho-STAT1 (Cell signaling 7649, 1:1000) anti-phospho-STAT2 (Cell signaling 88410; 1:1000) or 1% milk in 1x TBST: ⍺-A3A primary antibody [15] (1:1000 dilution), ⍺-tubulin primary antibody (Abcam 4074; 1:20000 dilution) at 4°C overnight (~16 hrs). Following washing in 1x TBS-T, blots were incubated in secondary antibody (HRP-Mouse secondary antibody (1:10000 dilution) or HRP-rabbit secondary antibody (1:20000 dilution)) for 1 hour at room temperature. A3A blots were developed with SuperSignal West Femto Maximum sensitivity substrate (Thermo scientific), all other blots were developed with ECL prime western blotting detecting reagent (Cytiva). Imaging was performed using a BioRad Chemidoc MP Imaging System using either chemi, or chemi-high-sensitivity setting.

### Quantification of A3A westerns

Intensity of anti-A3A and anti-tubulin signal from western blots of breast cancer cell line extracts in Figs 1 and S1 was obtained using ImageLab software following digital capture on a BioRad Chemidoc MP. Bands on each blot were detected using the sensitivity setting. Each blot contained extract from BT474 cells to serve as a control to standard relative expression. Briefly, band intensity volumes of A3A and tubulin in each cell line were divided by the respective volume in BT474 on the corresponding blot. Relative A3A values were subsequently divided by relative tubulin values to obtain a relative A3A expression standardized for loading. This value was subsequently multiplied by the ratio of A3A signal to tubulin signal from a single blot to obtain standardized A3A expression relative to tubulin for each cell line assessed.

### Cytidine deaminase assays

The amount of cellular cytidine deaminase activity in breast cancer cell lines was determined using a hairpin oligonucleotide (oTM-814) as in [14,50]. Briefly, 5 μg of cell extract generated in M-PER buffer was incubated with 1 μM oligonucleotide substrate in 20 mM Tris–HCl, pH 7.5, 1 mM DTT, 1 mM EDTA and for 18 hours at 37°C. Cytidine deamination was stopped by addition of Proteinase K (0.07 mg/ml Proteinase K and 10 mM Tris–HCl, pH 8, 1 mM EDTA, 0.5% SDS final concentration) and incubated at 37°C for 20 min. NaOH (0.1 M final) and formamide loading buffer (47.5% formamide, 9 mM EDTA, 0.0125% SDS final) were added prior to heating samples 95°C for 10 min to cleave abasic sites derived from UNG2 activity on deoxyuridine in the substrate. Substrate and products were separated on 1× TBE, 15% polyacrylamide gels with 7.9 M urea. Gels were imaged on a ChemiDoc MP using the Cy5 imaging settings.

### Statistical and bioinformatics analyses

All statistical analyses were performed in GraphPad Prism (version 9.3.1). Three biological replicates were analyzed for each experimental condition tested. Changes in gene expression were analyzed by ratio paired t-test. Correlations between relative qRT-PCR values, protein abundances, number of APOBEC signature mutations, cytidine deaminase activity, and RSEM normalized expression data were performed using Pearson correlation analysis. The minimum number estimate of APOBEC-induced mutations in breast cancer cell lines was determined as in [12] using mutation calls obtained from the Cancer Cell Encyclopedia. Unsupervised hierarchical gene expression clustering of RNA sequencing data from TCGA profiled breast adenocarcinomas [33] was conducted using Cluster 2.0 under default settings. The results were analyzed and visualized utilizing TreeView 2.0. A single node of 66 genes co-expressed with A3A was obtained by tree cutting the hierarchical clustering output. Genes within the node were input into String-db [51] (https://string-db.org) to generate a network diagram and for GO analysis. Heat maps comparing ATAC-seq data and A3A expression were produced using UCSC Xena (https://xenabrowser.net/heatmap/) [52]. Cell line ATAC-seq data at the *APOBEC3A* promoter was visualized using Integrative Genomics Viewer (https://igv.org/app/) [53]. Likely transcription factors regulating the *APOBEC3A* gene in primary breast cancers were identified by inputting the 66 genes expressed similarly to A3A into PASTAA [44] (from the Max Planck Institute for Molecular Genetics: https://trap.molgen.mpg.de/PASTAA.htm).

## Supporting information

S1 Table List of genes co-expressed with A3A in TCGA sequenced primary breast cancers. (XLSX)

S2 Table Transcription factor binding sites located in ATAC-seq peak within the APOBEC3A promoter proximal to the transcription start site. (XLSX)

S3 Table Top Genes for BACH1 regulation in breast cancer cells by PASTAA analysis. (XLSX)

S4 Table List of oligonucleotides used in this study. (XLSX)

S5 Table Numerical values for graphed data. (XLSX)

S1 Fig Correlation of A3A mRNA (A) and protein abundance (B) with cellular cytidine deaminase activity (% substrate cleavage) for cell lines listed in Fig 1A. The strength and significance of the correlations was determined by Pearson correlation test. Linear regression of the data is indicated by the solid black line. % substrate cleavage values for AU565, BT474, CAMA-1, HCC70, HCC202, MCF7, MDA-MB-361, MDA-MB-453, SKBR3, and T47D cells were obtained from [14].
(PDF)

S2 Fig RSEM gene-normalized mRNA-seq expression data from individual BRCA tumors correlating A3A expression and STAT1 (A) and STAT2 (B). The dashed line in each graph is a linear regression, with ’r’ representing the Pearson correlation coefficient, along with its corresponding p-value. The grey shaded region represents the 95% confidence interval for each regression line.
(PDF)

S3 Fig Impacts of transcription factor inhibition or knockdown in BRCA cell lines. (A) AU565, T47D, and HCC1395 cell lines were treated with 0, 25, 50, 100, and 200 μg/mL of fludarabine for 72 hrs, then A3A mRNA levels were measured and normalized to *HPRT1* mRNA levels. (B) STAT1 mRNA and A3A mRNA levels normalized to *HPRT1* levels in MDA-MB-453 cells transduced with scramble shRNA construct and STAT1-targeting shRNA construct. An approximate 15-fold decrease in STAT1 expression (p-value <0.05) and a 2-fold decrease in A3A expression (p-value <0.01). (C) STAT2 mRNA and A3A mRNA levels normalized to *HPRT1* mRNA in MDA-MB-453 cells transduced with scramble shRNA construct and STAT2-targeting shRNA construct. Approximately 7-fold reduced STAT2 expression (p-value <0.05) and a 10-fold reduced A3A expression (p-value >0.01) occurred. (D) RelA mRNA and A3A mRNA levels normalized to *HPRT1* levels in MDA-MB-453 transduced with scramble shRNA construct and Rel-A-targeting shRNA construct. A 60-fold decrease in Rel-A expression (p-value <0.05) and 25-fold decrease in A3A expression (p-value <0.05) occurred. All measurements show the mean value and standard deviation for 3 independent biological measurements.
(PDF)

S4 Fig STAT2 shRNA knockdown in BT474 cells. (A) STAT2 mRNA levels normalized to *HPRT* levels in BT474 cell line transduced with scramble shRNA construct and STAT2-targeting shRNA construct. Approximate 2-fold decrease in STAT2 expression; significant with p-value <0.05
(PDF)

S5 Fig STAT1/2 phosphorylation in breast cancer cells. (A) Western analysis of STAT1, phospho-STAT1 (pSTAT1), STAT2, phospho-STAT2 (pSTAT2), A3A, and tubulin in a panel of breast cancer cell lines. Phospho-STAT1/2 occurs only at very low levels and does not correlate with A3A protein abundance. (B) STAT1/2 signaling is canonically established by JAK-mediated phosphorylation of STAT1 or STAT2. qRT-PCR assessment of JAK inhibitors (ruxolitinib and pacritinib) impact on A3A mRNA levels in BT474 and MDA-MB-453 cells.
(PDF)

S6 Fig Basal Bach1 and A3A mRNA expression in breast cancer cells. RSEM-normalized RNA-seq expression of *BACH1* (log_10_ transformed) was compared to qRT-PCR measured A3A mRNA relative to *HPRT1* (log_10_ transformed) for cells in Fig 1A. Correlation analysis comparing co-expression was assessed using Pearson correlation test. Linear regression is indicated by solid black line.
(PDF)
