# Supplementary material for: Her2 amplification, Rel-A, and Bach1 can influence APOBEC3A expression in breast cancer cells
Source: PLoS Genet. 2024 May 28;20(5):e1011293. doi: 10.1371/journal.pgen.1011293 (PMC11161071; doi:10.1371/journal.pgen.1011293)
Supplement: S1 Fig — Correlation of A3A mRNA (A) and protein abundance (B) with cellular cytidine deaminase activity (% substrate cleavage) for cell lines listed in Fig 1A. The strength and significance of the correlations was determined by Pearson correlation test. Linear regression of the data is indicated by the solid black line. % substrate cleavage values for AU565, BT474, CAMA-1, HCC70, HCC202, MCF7, MDA-MB-361, MDA-MB-453, SKBR3, and T47D cells were obtained from [14]. (PDF) [file pgen.1011293.s006.pdf]

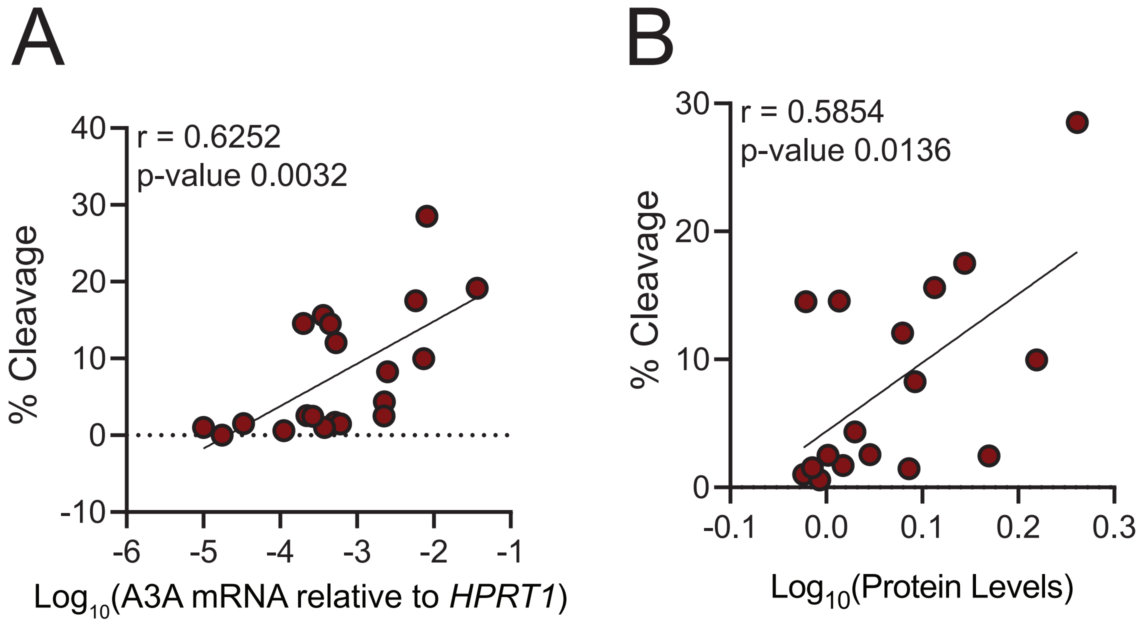

S1 Fig: Correlation of A3A mRNA (A) and protein abundance (B) with cellular cytidine deaminase activity (% substrate cleavage) for cell lines listed in Figure 1A. The strength and significance of the correlations was determined by Pearson correlation test. Linear regression of the data is indicated by the solid black line. % substrate cleavage values for AU565, BT474, CAMA-1, HCC70, HCC202, MCF7, MDA-MB-361, MDA-MB-453, SKBR3, and T47D cells were obtained from [15].
