# Supplementary material for: Her2 amplification, Rel-A, and Bach1 can influence APOBEC3A expression in breast cancer cells
Source: PLoS Genet. 2024 May 28;20(5):e1011293. doi: 10.1371/journal.pgen.1011293 (PMC11161071; doi:10.1371/journal.pgen.1011293)
Supplement: S2 Fig — RSEM gene-normalized mRNA-seq expression data from individual BRCA tumors correlating A3A expression and STAT1 (A) and STAT2 (B). The dashed line in each graph is a linear regression, with ’r’ representing the Pearson correlation coefficient, along with its corresponding p-value. The grey shaded region represents the 95% confidence interval for each regression line. (PDF) [file pgen.1011293.s007.pdf]

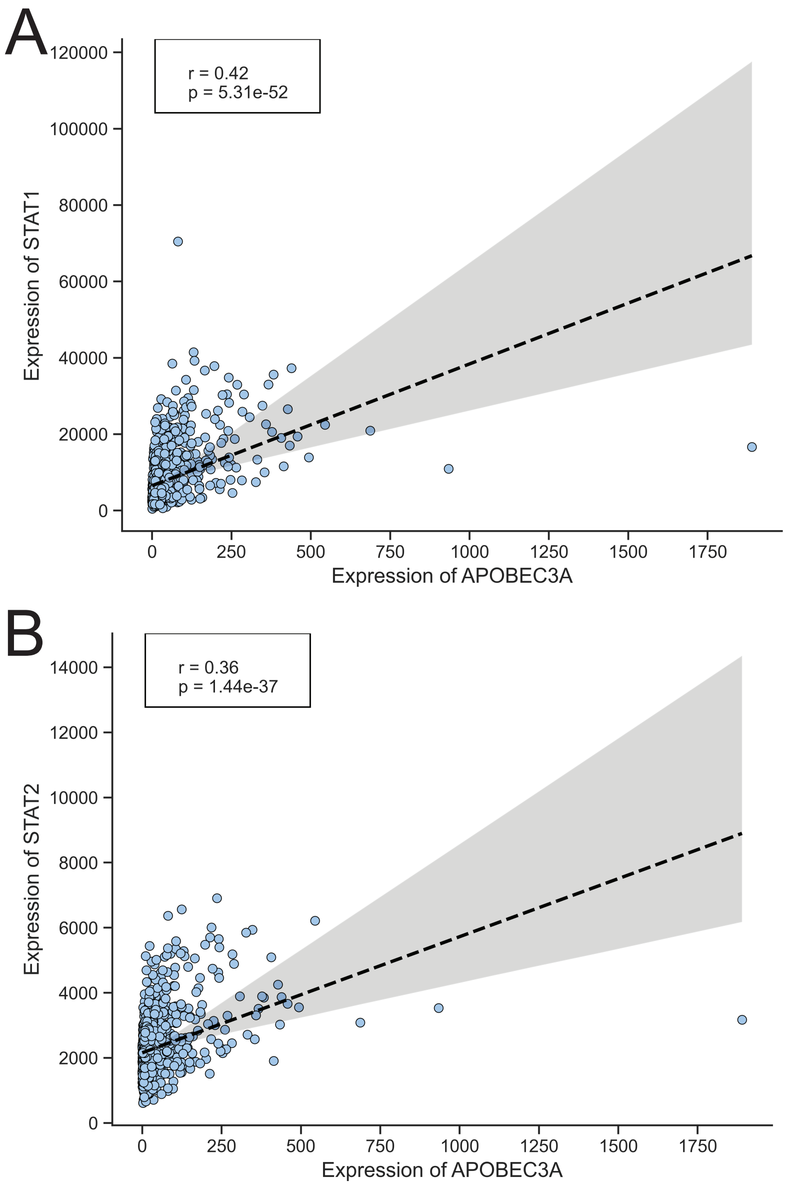

S2 Fig: RSEM gene-normalized mRNA-seq expression data from individual BRCA tumors correlating A3A expression and STAT1 (A) and STAT2 (B). The dashed line in each graph is a linear regression, with 'r' representing the Pearson correlation coefficient, along with its corresponding p-value. The grey shaded region represents the 95% confidence interval for each regression line.
