# Supplementary material for: Her2 amplification, Rel-A, and Bach1 can influence APOBEC3A expression in breast cancer cells
Source: PLoS Genet. 2024 May 28;20(5):e1011293. doi: 10.1371/journal.pgen.1011293 (PMC11161071; doi:10.1371/journal.pgen.1011293)
Supplement: S3 Fig — (A) AU565, T47D, and HCC1395 cell lines were treated with 0, 25, 50, 100, and 200 μg/mL of fludarabine for 72 hrs, then A3A mRNA levels were measured and normalized to HPRT1 mRNA levels. (B) STAT1 mRNA and A3A mRNA levels normalized to HPRT1 levels in MDA-MB-453 cells transduced with scramble shRNA construct and STAT1-targeting shRNA construct. An approximate 15-fold decrease in STAT1 expression (p-value <0.05) and a 2-fold decrease in A3A expression (p-value <0.01). (C) STAT2 mRNA and A3A mRNA levels normalized to HPRT1 mRNA in MDA-MB-453 cells transduced with scramble shRNA construct and STAT2-targeting shRNA construct. Approximately 7-fold reduced STAT2 expression (p-value <0.05) and a 10-fold reduced A3A expression (p-value >0.01) occurred. (D) RelA mRNA and A3A mRNA levels normalized to HPRT1 levels in MDA-MB-453 transduced with scramble shRNA construct and Rel-A-targeting shRNA construct. A 60-fold decrease in Rel-A expression (p-value <0.05) and 25-fold decrease in A3A expression (p-value <0.05) occurred. All measurements show the mean value and standard deviation for 3 independent biological measurements. (PDF) [file pgen.1011293.s008.pdf]

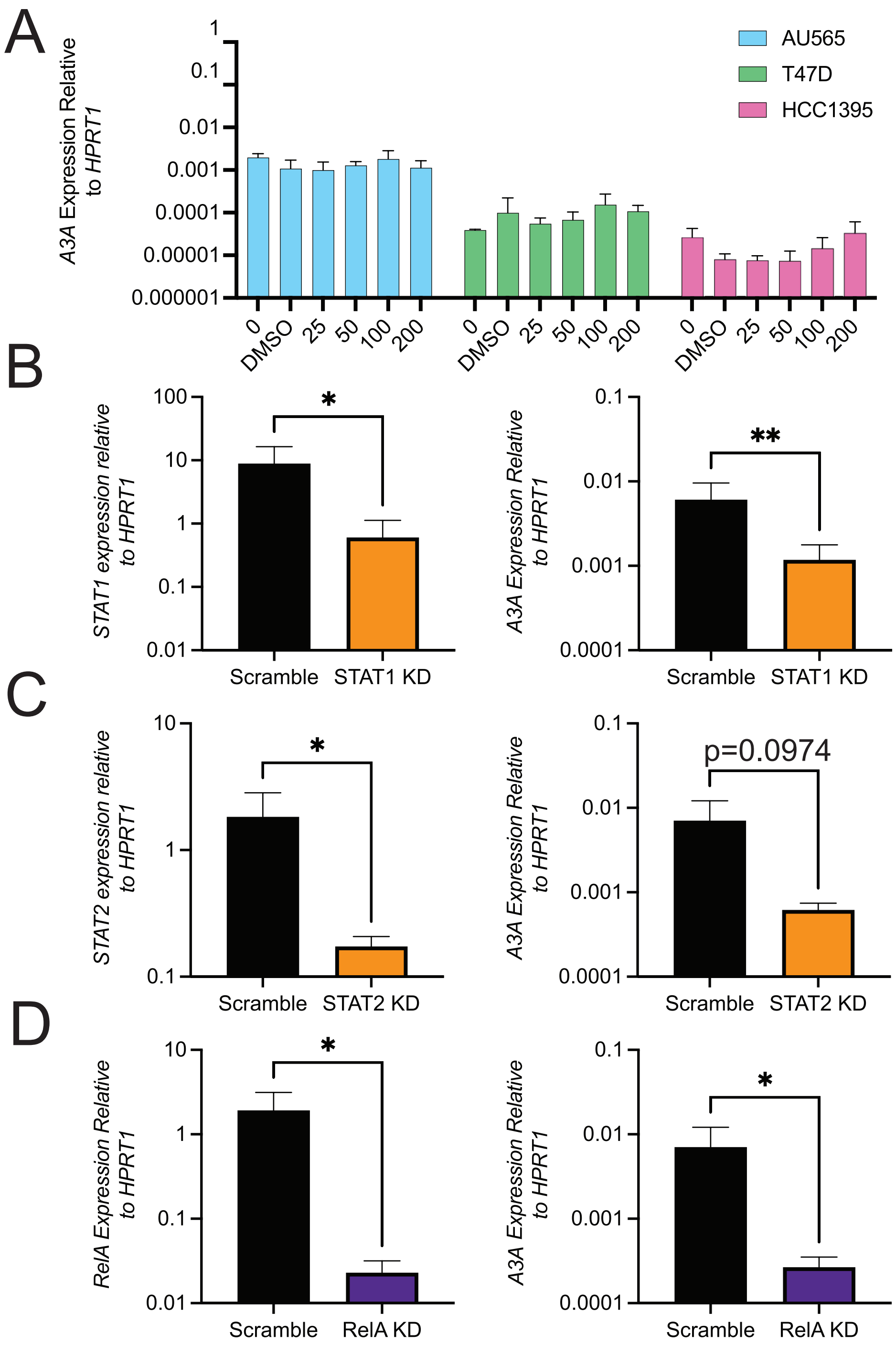


**S3 Fig:** Impacts of transcription factor inhibition or knockdown in BRCA cell lines. (A) AU565, T47D, and HCC1395 cell lines were treated with 0, 25, 50, 100, and 200 µg/mL of fludarabine for 72 hrs, then A3A mRNA levels were measured and normalized to HPRT1 mRNA levels. (B) STAT1 mRNA and A3A mRNA levels normalized to HPRT1 levels in MDA-MB-453 cells transduced with scramble shRNA construct and STAT1-targeting shRNA construct. An approximate 15-fold decrease in STAT1 expression (p-value <0.05) and a 2-fold decrease in A3A expression (p-value <0.01). (C) STAT2 mRNA and A3A mRNA levels normalized to HPRT1 mRNA in MDA-MB-453 cells transduced with scramble shRNA construct and STAT2-targeting shRNA construct. Approximately 7-fold reduced STAT2 expression (p-value <0.05) and a 10-fold reduced A3A expression (p-value >0.01) occurred. (D) RelA mRNA and A3A mRNA levels normalized to HPRT1 levels in MDA-MB-453 transduced with scramble shRNA construct and Rel-A-targeting shRNA construct. A 60-fold decrease in Rel-A expression (p-value <0.05) and 25-fold decrease in A3A expression (p-value <0.05) occurred. All measurements show the mean value and standard deviation for 3 independent biological measurements.
