# Supplementary material for: Her2 amplification, Rel-A, and Bach1 can influence APOBEC3A expression in breast cancer cells
Source: PLoS Genet. 2024 May 28;20(5):e1011293. doi: 10.1371/journal.pgen.1011293 (PMC11161071; doi:10.1371/journal.pgen.1011293)
Supplement: S4 Fig — (A) STAT2 mRNA levels normalized to HPRT levels in BT474 cell line transduced with scramble shRNA construct and STAT2-targeting shRNA construct. Approximate 2-fold decrease in STAT2 expression; significant with p-value <0.05 (PDF) [file pgen.1011293.s009.pdf]

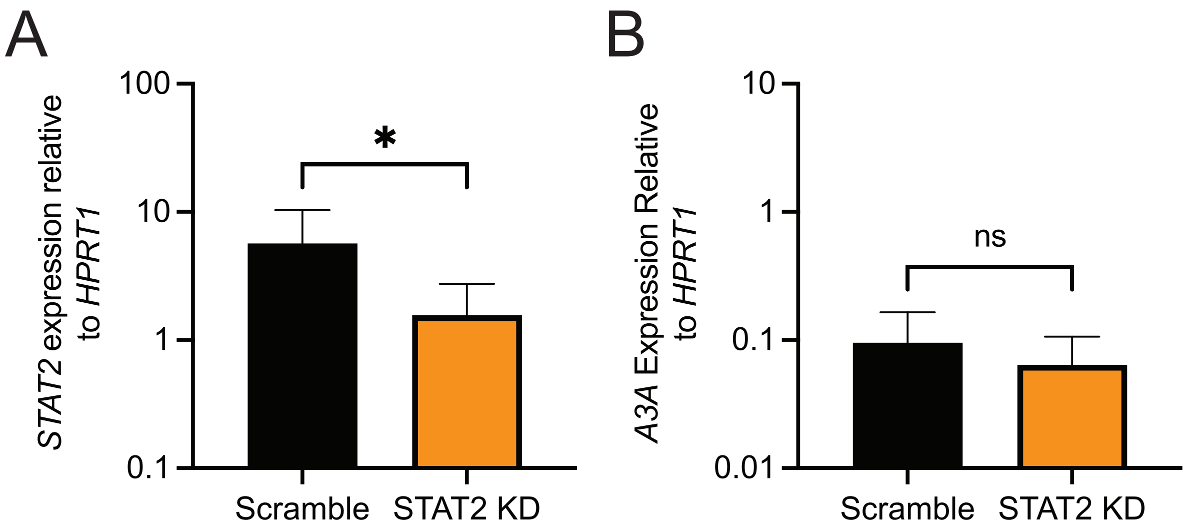

S4 Fig: STAT2 shRNA knockdown in BT474 cells. (A) STAT2 mRNA levels normalized to HPRT levels in BT474 cell line transduced with scramble shRNA construct and STAT2-targeting shRNA construct. Approximate 2-fold decrease in STAT2 expression; significant with p-value <0.05
