# Supplementary material for: Her2 amplification, Rel-A, and Bach1 can influence APOBEC3A expression in breast cancer cells
Source: PLoS Genet. 2024 May 28;20(5):e1011293. doi: 10.1371/journal.pgen.1011293 (PMC11161071; doi:10.1371/journal.pgen.1011293)
Supplement: S5 Fig — (A) Western analysis of STAT1, phospho-STAT1 (pSTAT1), STAT2, phospho-STAT2 (pSTAT2), A3A, and tubulin in a panel of breast cancer cell lines. Phospho-STAT1/2 occurs only at very low levels and does not correlate with A3A protein abundance. (B) STAT1/2 signaling is canonically established by JAK-mediated phosphorylation of STAT1 or STAT2. qRT-PCR assessment of JAK inhibitors (ruxolitinib and pacritinib) impact on A3A mRNA levels in BT474 and MDA-MB-453 cells. (PDF) [file pgen.1011293.s010.pdf]

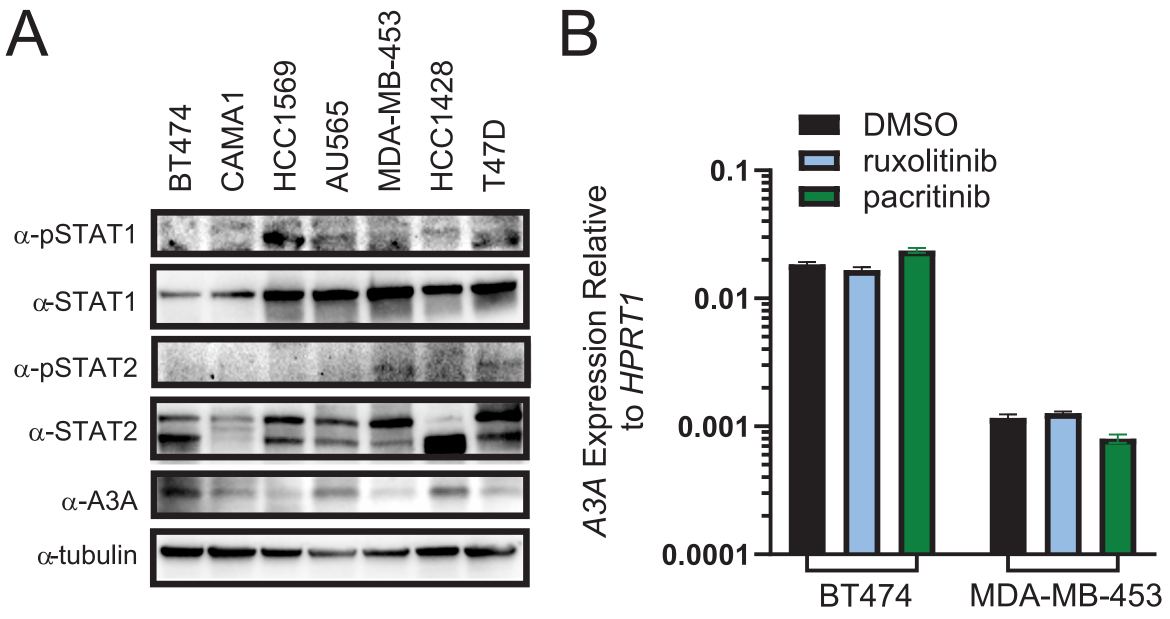

S5 Fig: STAT1/2 phosphorylation in breast cancer cells. (A) Western analysis of STAT1, phospho-STAT1 (pSTAT1), STAT2, phospho-STAT2 (pSTAT2), A3A, and tubulin in a panel of breast cancer cell lines. Phospho-STAT1/2 occurs only at very low levels and does not correlate with A3A protein abundance. (B) STAT1/2 signaling is canonically established by JAK-mediated phosphorylation of STAT1 or STAT2. qRT-PCR assessment of JAK inhibitors (ruxolitinib and pacritinib) impact on A3A mRNA levels in BT474 and MDA-MB-453 cells.
