# Supplementary material for: Her2 amplification, Rel-A, and Bach1 can influence APOBEC3A expression in breast cancer cells
Source: PLoS Genet. 2024 May 28;20(5):e1011293. doi: 10.1371/journal.pgen.1011293 (PMC11161071; doi:10.1371/journal.pgen.1011293)
Supplement: S6 Fig — RSEM-normalized RNA-seq expression of BACH1 (log10 transformed) was compared to qRT-PCR measured A3A mRNA relative to HPRT1 (log10 transformed) for cells in Fig 1A. Correlation analysis comparing co-expression was assessed using Pearson correlation test. Linear regression is indicated by solid black line. (PDF) [file pgen.1011293.s011.pdf]

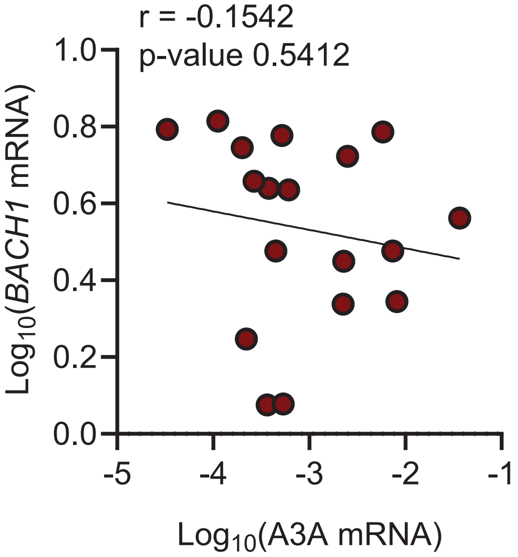

S6 Fig: Basal Bach1 and A3A mRNA expression in breast cancer cells. RSEM-normalized RNA-seq expression of BACH1 (log10 transformed) was compared to qRT-PCR measured A3A mRNA relative to HPRT1 (log10 transformed) for cells in Figure 1A. Correlation analysis comparing co-expression was assessed using Pearson correlation test. Linear regression is indicated by solid black line.
